# Lung mechanics in juvenile and adult *Chelonoidis carbonarius*

**DOI:** 10.1242/jeb.247852

**Published:** 2024-10-10

**Authors:** Paulo Roberto Custodio de Oliveira, Pedro Trevizan-Baú, Ray Brasil Bueno de Souza, Wilfried Klein

**Affiliations:** ^1^ Departamento de Biologia, Faculdade de Filosofia, Ciências e Letras de Ribeirão Preto, Universidade de São Paulo, 14040-901 Ribeirão Preto, São Paulo, Brazil; ^2^ Instituto Federal do Paraná, Campus Avançado Goioerê, Goioerê, PR, 87360-000, Brazil

**Keywords:** Comparative respiratory physiology, Lung mechanics, Testudines

## Abstract

Testudines possess a rigid shell that influences the mechanics of the respiratory system. We studied respiratory mechanics in the terrestrial red-footed tortoise *Chelonoidis carbonarius* (Cryptodira), comparing juvenile individuals with a less ossified and more flexible carapace with adults with a well-ossified rigid shell. Combined with these ontogenetic differences, we analyzed respiratory system mechanics with animals in a supine and a prone position, as well as in the isolated lungs, to evaluate the impact of the viscera on breathing mechanics. To do so, we used established protocols to measure pulmonary volume (i.e. resting, *V*_Lr_; and maximum, *V*_Lm_), static (*C*_stat_) and dynamic (*C*_dyn_) compliance, and the work of breathing (*W*). We observed that isolated lungs displayed increased *V*_Lr_, *V*_Lm_, *C*_stat_ and *C*_dyn_ and decreased *W*. Additionally, pulmonary volume, compliance and *W* were affected by evaluated position, such as a smaller *V*_Lr_ in a supine position. *C*_dyn_ and *W* showed a volume dependency while frequency had less influence on these variables. At similar levels of ventilation, juveniles showed a lower *W* than adults when standardized by body mass, but similar *W* when standardized by *V*_Lr_. Clear ontogenetic changes could be observed in breathing mechanics between juvenile and adult *C. carbonarius*. While these differences might largely be explained by variation in shell ossification, other explanations such as differences in visceral proportions or developmental degree of the post-pulmonary septum should also be taken into account.

## Teaser

**Summary:** Analysis of respiratory system mechanics in juvenile and adult terrestrial red-footed tortoise shows positional and ontogenetic effects on mechanical properties, shedding light on how life stage may influence respiratory function.

## INTRODUCTION

Among reptiles, Testudines show unique anatomical and morphological features that make them interesting models for studying breathing mechanics. In addition to multichambered lungs (Perry, 1998), Testudines present ribs fused with the spine and loss of intercostal muscles, as well as the presence of a bony plastron (Lyson et al., 2014). Consequently, Testudines present a rigid shell that impedes lung ventilation by the mechanisms seen in other amniotes and, thus, fundamentally alters the mechanics of the respiratory system (Jackson, 1971; Vitalis and Milsom, 1986a). Turtles, tortoises and terrapins use abdominal muscles to promote changes in intrapulmonary pressure during ventilatory activities (Gans and Hughes, 1967; Gaunt and Gans, 1969; Jackson, 1971; Lambertz, et al., 2010; Lyson et al., 2014). Additionally, their multichambered lungs are connected to the dorsal shell through mesopneumonia or may be fused directly to the carapace, while ventral mesopneumonia, or an associated structure named the post-pulmonary septum (PPS; Duncker, 1978; Perry, 1998; Perry and Sander, 2004), connect the lungs to the ventrally located viscera (Broman, 1904, 1937; Lambertz et al., 2010). Similar to all the anatomical and morphological features, the presence of the PPS in the red-eared slider *Trachemys scripta* has recently been demonstrated to significantly influence lung mechanics (Souza and Klein, 2021).

In terms of lung ventilation, Testudines, like other reptiles, show an intermittent ventilatory pattern, by which single breaths or burst of breaths are separated by non-ventilatory periods of varied duration (Glass and Wood, 1983; Vitalis and Milsom, 1986a). Ultimately, understanding the breathing pattern implies observing the relationship between ventilation, gas exchange and ventilatory mechanics (Pages et al., 1990). Importantly, ventilatory mechanics plays a crucial role in lung ventilation, influencing breathing frequency and tidal volume, as well as the cost to ventilate the lungs (Perry and Duncker, 1980; Milsom and Vitalis, 1984; Vitalis and Milsom, 1986a,b; Pages et al., 1990; Perry, 1998; Reichert et al., 2019).

To our knowledge, the mechanical properties of ventilation in Testudines have mostly been investigated in aquatic or semi-aquatic species, including *T. scripta* (Jackson, 1971; Vitalis and Milsom, 1986a,b; Lee and Milsom, 2016; Souza and Klein, 2021), *Caretta caretta* (Lutcavage et al., 1989) and *Mauremys caspica* (Pages et al., 1990). These studies analyzed lung volume, static and dynamic compliance and/or the work of breathing. Jackson (1971) indicated that the unique pulmonary mechanics of *T. scripta* enables them to adjust pulmonary volume and maintain constant pressure with little or no muscular effort (low cost of breathing). Lutcavage et al. (1989) and Williams et al. (2021) studied static compliance and lung volume in prone and supine positions in non-terrestrial turtles and observed alterations in lung mechanics depending on the position of the animal. The present study is the first that aims to describe lung mechanics in the terrestrial red-footed tortoise *Chelonoidis carbonarius*, including both juvenile and adult individuals. A recent study compared mechanical properties of the respiratory system in juvenile and adult *Caiman yacare* and found differences in lung compliance, indicating that the morphological and anatomical changes during development lead to a reduction in body wall compliance (Reichert et al., 2019). Therefore, because of their less ossified shell (Oliveira et al., 2023), we hypothesize that juvenile *C. carbonarius* might also present greater respiratory system compliance compared with adult animals. To test our working hypothesis, we studied lung mechanics in both juvenile and adult *C. carbonarius*. Specifically, we describe and compare pulmonary volume, static and dynamic compliance and the work of breathing among age groups. Furthermore, we also investigated these variables in both supine and prone positions, as well as within the lungs alone, to understand the role of visceral pressure on lung mechanics. This latter point is based on the widespread practice to study respiratory system mechanics with animals in supine position. In Testudines, the lungs are placed dorsally within the body cavity, receiving the full visceral mass when supine, but not prone, a more natural position.

## MATERIALS AND METHODS

### Animals

Juveniles (*n*=4, mean±s.e.m. body mass 0.08±0.01 kg) and adults (*n*=10, 3.25±0.89 kg) *Chelonoidis carbonarius* (Spix 1824) were obtained from Jacarezário of the Universidade Estadual Paulista Júlio Mesquita Filho (UNESP) in Rio Claro (São Paulo, Brazil) during 2014 and 2015. The experimental animals were transferred to the local animal care facility at the Faculdade de Filosofia, Ciências e Letras de Ribeirão Preto (São Paulo, Brazil), where they were housed for 3 months until experiments were performed. All animals were housed under a 12 h:12 h light:dark cycle, in a controlled environment of 25°C. They were fed 3 times a week with fruit and vegetables and provided with water *ad libitum*. All experimental procedures were conducted under license SISBIO (35221-1 and 35221-8) and protocol CEUA-FFCLRP-Campus de Ribeirão Preto (12.1.1541.53.0 and 16.5.835.59.0).

### Experimental setup and data analysis

The animals were euthanized through an intraperitoneal injection of thiopental (150 mg kg^−1^) and lidocaine (5 mg kg^−1^), and the trachea was then cannulated and connected using a three-way valve to a syringe and a pressure transducer (DELTRAN). The pressure transducer signal was amplified (AECAD 04P) and recorded using a data acquisition system (PowerLab 8/35), while data were analyzed using Labchart 7.0 software (AdInstruments).

To obtain the resting pulmonary volume (*V*_Lr_) and maximum pulmonary volume (*V*_Lm_), we followed the experimental protocol proposed by Perry and Duncker (1978) and Klein et al. (2003). Starting with the respiratory system equilibrated with atmospheric pressure, air was removed from the lungs in a stepwise manner using a syringe until reaching a pressure of −10 cmH_2_O, giving *V*_Lr_. Afterwards, air was added in a stepwise manner until reaching a pressure of +20 cmH_2_O to obtain *V*_Lm_. Lastly, air was removed again in steps to return to the initial state of inflation. This procedure enabled us to generate the volume–pressure curves both for inflation and deflation of the respiratory system. This experiment was performed in all the animals.

To determine the static compliance (*C*_stat_), we followed Perry and Duncker (1978) and Vitalis and Milsom (1986b). Using the inflation volume–pressure curve, we obtained the volume and pressure values of the most inclined part of the inflation curve, and calculated *C*_stat_ by applying the following formula:
(1)

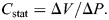
After acquiring lung volume and *C*_stat_, we measured dynamic compliance (*C*_dyn_) by replacing the syringe with a pneumotachograph, which in turn was ventilated through a ventilation pump (Inspira, Harvard Apparatus). Before each *C*_dyn_ measurement, the respiratory system was fully inflated and allowed to deflate to *V*_Lr_. Known volumes of air that were used in this study (0.1, 0.3, 0.6, 0.9, 1.2, 1.5, 2, 3 and 4 ml in juveniles; 5, 10, 15, 20, 25 and 30 ml in adults) were applied at different frequencies (frequencies used for both groups: 5, 10, 15, 20, 25 (except for adults), 30, 40 and 50 min^−1^). These volumes or air and pump frequencies were chosen based on our previous study showing tidal volumes and breathing frequencies of both juvenile and adult *C. carbonarius* (Trevizan-Baú et al., 2018; Oliveira et al., 2023). However, it is important to note that we also applied non-natural volumes and frequencies, so that we could challenge the respiratory system at its limits, obtaining a better understanding of this species' breathing mechanics, especially regarding dynamic compliance and work of breathing. Then, the pressure–volume loops generated were used to calculate *C*_dyn_ and work of breathing (*W*). *C*_dyn_ was obtained by calculating the slope of the line connecting the points of zero flow on the loop (Milsom and Vitalis, 1984; Vitalis and Milsom, 1986b; York et al., 2017). According to Vitalis and Milsom (1986b), the total *C*_dyn_ in the respiratory system is the sum of compliance performed by both the lung and body wall. Thus, to find the compliance of the body wall (*C*_B_), we used the following equation:
(2)

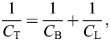
where *C*_L_ represents the compliance of the isolated lungs and *C*_T_ the compliance of the intact respiratory system.

*W* was calculated as the area of the pressure–volume loop delimitated by the inflation curve, the *y*-axis, and by a horizontal line connecting the *y*-axis to the end-inflation point (Vitalis and Milsom, 1986b; York et al., 2017). The area was obtained by using the software ImageJ to determine the number of pixels in a given area and transformed into ml cmH_2_O by determining the number of pixels in an area given by 1 ml and 1 cmH_2_O. The relationship between the work and the frequency (min^−1^) was used to reach the minute work (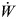
in ml cmH_2_O min^−1^ kg^−1^). Following Souza and Klein (2021), linear regressions were employed, utilizing log_10_-transformed work values standardized by body mass (log_10_*W*/*M*_B_) against log_10_-transformed pump ventilation (log_10_*V*_P_), with data segregated based on varying pump frequencies (*f*) to identify isopleths. Vertical lines corresponding to pump ventilations of 1, 5, 10, 25, 50, 100, 200, 300, 400, 500, 750 and 1000 ml min^−1^ were depicted on the same graph. The anti-log of the work values for each of these lines intersecting the isopleths was used to calculate minute work (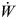), which was then plotted against respiratory frequency, with data segregated according to different ventilation levels.

All described procedures were performed in three different conditions: animals in supine position, followed by prone position, and lastly with the viscera removed in order to expose the lungs, but with the PPS maintained intact. The exposed lungs were humidified with saline solution (0.9%) during the entire procedure. In supine and prone positions, care was taken that neither the limbs nor the head was retracted into the carapace.

### Statistical analysis

Data analysis was performed with R 4.2.1 (http://www.R-project.org/), and for graphic construction we used the software GraphPad Prism 5.0 (https://www.graphpad.com/). The relationship between the variables in all tested conditions and between the groups was determined by applying a generalized model for repeated measures (Generalized Estimating Equations, GEE). Models with Gamma and Gaussian distribution were compared with quasi-information criterion (QIC) and correlation information criterion (CIC) tests, along with adjustment of the residuals of each model to a Gaussian *Q*–*Q* plot, to choose the best-fitting model. The relationship between *C*_stat_ and body size was plotted in a log–log relationship with *M*_B_ to obtain an allometric equation. The pairwise comparison of slopes through linear regression was conducted in R to assess whether the slopes in different combinations exhibited significant differences. *P*-values ≤0.05 were considered significant.

## RESULTS

### Lung volume and static compliance

**Fig. 1.**
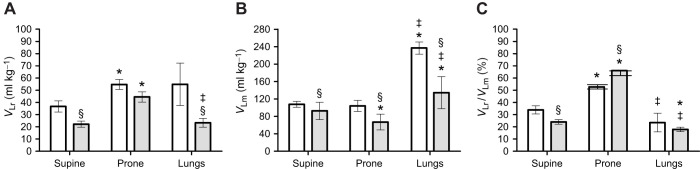
**Lung volume in *Chelonoidis carbonarius* in supine and prone positions and in isolated lungs.** Body mass-standardized resting (*V*_Lr_; A) and maximum (*V*_Lm_; B) lung volume, and their relationship *V*_Lr_/*V*_Lm_ (C) in juveniles (white) and adults (gray). Data are means±s.e.m. *Significant difference (*P*<0.05) from the supine position; ^‡^significant difference (*P*<0.05) from the prone position; ^§^significant difference (*P*<0.05) between juveniles and adults.

Pulmonary volume was significantly affected by the test conditions (supine, prone and isolated) as well as life stage (Fig. 1). *V*_Lr_ was significantly greater in the prone position in adults and juveniles. However, *V*_Lr_ in juveniles in isolated lungs was similar to that in the prone position, and *V*_Lr_ in adults in isolated lungs was similar to *V*_Lr_ in the supine position. Also, *V*_Lr_ in adults was significantly decreased in isolated lungs and in the supine position compared with that in juveniles. *V*_Lm_ was significantly augmented in isolated lungs when compared with that in supine and prone positions in adults and juveniles, and the lowest *V*_Lm_ was observed in adults in the prone position. In all three tested conditions, *V*_Lm_ was significantly smaller in adults when compared with juveniles. Analyzing the relationship *V*_Lr_*/V*_Lm_, we observed that *V*_Lr_ contributed significantly more to *V*_Lm_ in the prone position, being greatest in adults. Compared with juveniles, adults showed a significantly greater *V*_Lr_*/V*_Lm_ in the prone position and a significantly lower *V*_Lr_*/V*_Lm_ in the supine position. *V*_Lr_*/V*_Lm_ was lowest in the isolated lungs.

**Fig. 2.**
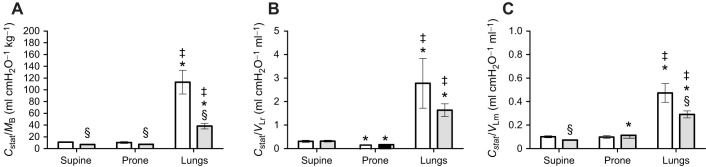
**Static compliance (*C*_stat_) in *C. carbonarius* in supine and prone positions and in isolated lungs.** (A) *C*_stat_ standardized by body mass (*M*_B_), (B) *C*_stat_ standardized by *V*_Lr_ and (C) *C*_stat_ standardized by *V*_Lm_ in juveniles (white) and in adults (gray). Data are means±s.e.m. *Significant difference (*P*<0.05) from the supine position; ^‡^significant difference (*P*<0.05) from the prone position; ^§^significant difference (*P*<0.05) between juveniles and adults.

Next, *C*_stat_ was standardized by *M*_B_, *V*_Lr_ and *V*_Lm_ (Fig. 2). We observed that *C*_stat_/*M*_B_ was not significantly different between supine and prone positions, but adults showed significantly lower *C*_stat_ in all tested conditions when compared with juveniles. Significant differences between prone and supine positions were found in *C*_stat_/*V*_Lr_ for both juveniles and adults, while for *C*_stat_/*V*_Lm_, only adults presented a higher compliance in the prone position. For the intact system (prone and supine), juveniles, when compared with adults, showed significantly higher *C*_stat_/*V*_Lm_ only in the supine condition. *C*_stat_ was greatest in the isolated lungs for all standardizations and significantly different between age groups when standardized by *M*_B_ and *V*_Lm_.

**Fig. 3.**
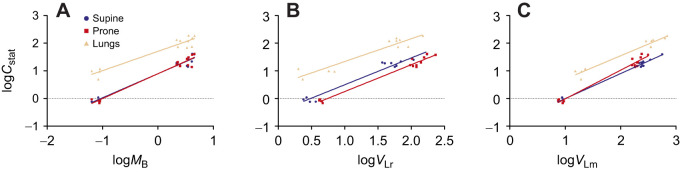
**Allometric relationship of log *C*_stat_ with log *M*_B_, log *V*_Lr_ and log *V*_Lm_ in *C. carbonarius* in supine and prone positions and in isolated lungs.** Graphs show log *C*_stat_ (ml cmH_2_O) versus log *M*_B_ (kg; A), log *V*_Lr_ (ml; B) and log *V*_Lm_ (ml; C). Blue diamonds represent supine position; red squares, prone position; and yellow triangles, isolated lungs.

Plotting non-standardized *C*_stat_ against *M*_B_, *V*_Lr_ and *V*_Lm_ gave the intraspecific regressions for each tested condition (Fig. 3). *C*_stat_ showed strong and significant correlation with *M*_B_ and pulmonary volume (Table 1).

**Table 1.**
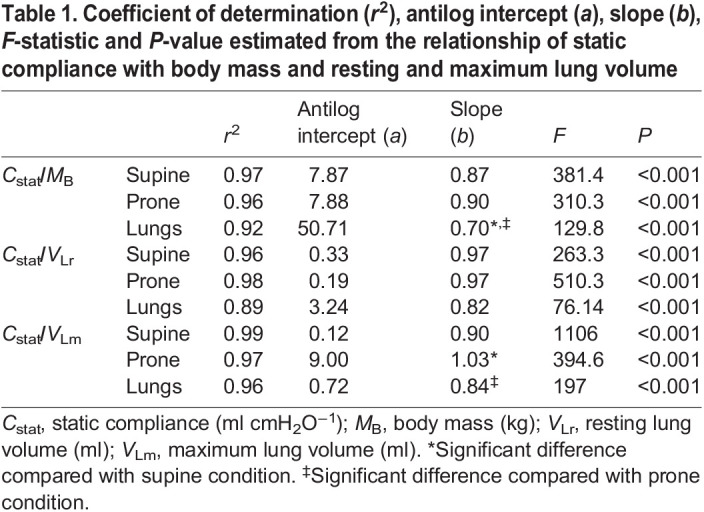
Coefficient of determination (*r*^2^), antilog intercept (*a*), slope (*b*), *F*-statistic and *P*-value estimated from the relationship of static compliance with body mass and resting and maximum lung volume

### Dynamic compliance

The position of the body for the intact respiratory system (supine and prone) showed a significant influence on *C*_dyn_, with juveniles presenting more differences between supine and prone positions at greater volumes (3 and 4 ml). The greatest *C*_dyn_ for the intact respiratory system was observed when we ventilated juveniles at the highest volume (4 ml). The isolated lungs, however, showed the greatest *C*_dyn_ in both age groups.

**Fig. 4.**
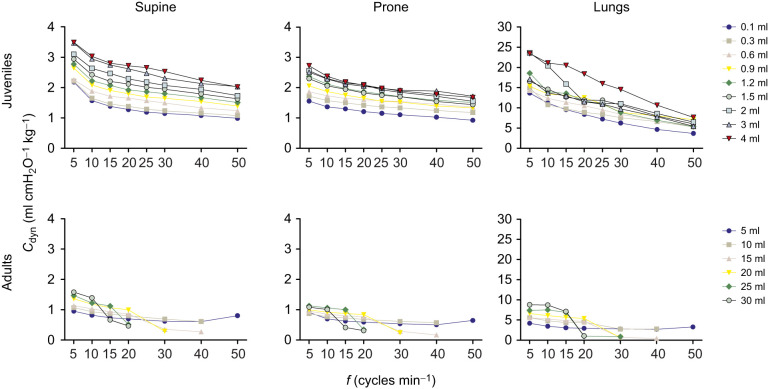
**Relationship between *M*_B_-standardized dynamic compliance (*C*_dyn_) and pump frequency (*f*) in juvenile (top) and adult (bottom) *C. carbonarius* in supine and prone positions and in isolated lungs.** Please note the different scale for the *y*-axis for the isolated lungs.

Juveniles exhibited a more predictable pattern regarding *C*_dyn_. We show in Fig. 4 that *C*_dyn_ decreased in all three conditions in a comparable manner with increasing ventilation frequency, while showing a tendency to increase with increasing pump volume at each given frequency. In adults, *C*_dyn_ was lower than in juveniles and showed a (volume-dependent) remarkable significant decrease between frequencies of 10 and 30 cycles min^−1^ (Fig. 4).

**Fig. 5.**
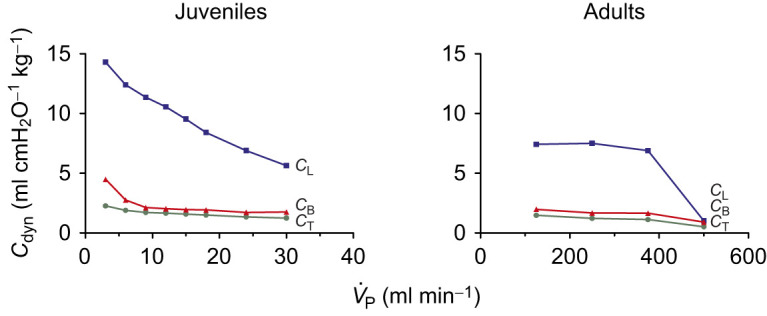
**Relationship of *C*_dyn_ in the lung, intact respiratory system (supine position; ‘total system’) and body cavity with pump ventilation (**
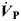
**) in juvenile (left) and adult (right) *C. carbonarius*.** *C*_L_, lung compliance; *C*_T_, total system compliance; *C*_B_, body cavity compliance. Data are means±s.e.m. Pump volumes of 0.6 ml (juveniles) and 25 ml (adults) were compared as a correction of these pump volumes by *M*_B_ yields very similar volumes of 7.5 ml kg^−1^ (juveniles) and 7.7 ml kg^−1^ (adults).

To better compare *C*_dyn_ between juveniles and adults, we decided to use pulmonary data standardized by *M*_B_. Thus, we chose pump volumes of 0.6 ml for juveniles and 25 ml for adults, as these volumes represent, respectively, ventilatory volumes of 7.5 and 7.7 ml kg^−1^. Plotting *C*_dyn_ in this way for the intact respiratory system, the isolated lungs and the values calculated for the body cavity against pump ventilation (Fig. 5) indicates the lungs are the most compliant component of these animals' respiratory system, showing a greater ventilation dependency in juveniles than in adults. Comparing juveniles with adults, lung compliance (*C*_L_) did not show statistically significant differences between the groups, but for the total system (*C*_T_) and the body cavity (*C*_B_), juveniles showed a higher compliance, especially when frequency increased.

### Work of breathing

**Fig. 6.**
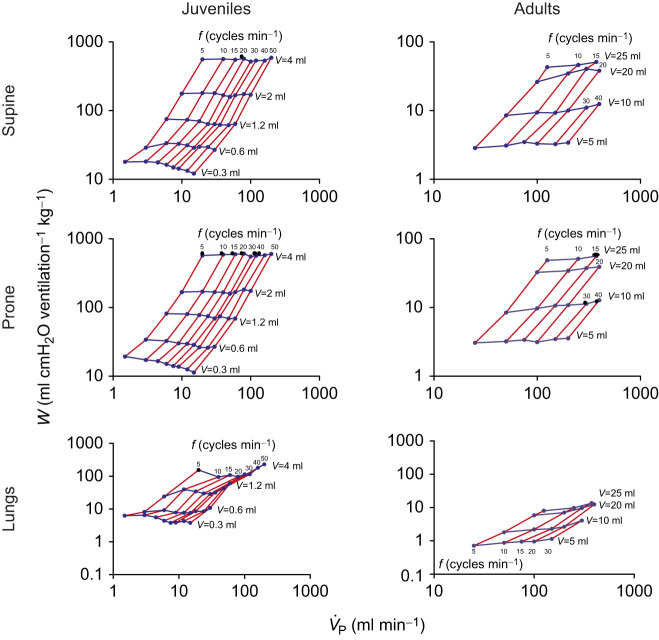
**Relationship between work of breathing (*W*) and total** 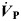 **in different combinations of tested volumes (*V*) and pump frequencies (*f*) for juvenile (left) and adult (right) *C.*** ***carbonarius*** **in supine (top) and prone position (middle) and in isolated lungs (bottom)**. Please note the different scales for juveniles and adults versus isolated lungs.

Fig. 6 shows that work per breath (*W*) was significantly impacted by lung volume, whereas frequency seemed to have less influence. However, it is important to note that an increase in frequency at the smallest lung volume (0.3 ml for juveniles, and 5 and 10 ml for adults) resulted in a slight decrease in *W* in both supine and prone positions for juveniles, while for adults it resulted in a slight increase in *W*. At the other volumes, *W* was virtually unaltered by increasing frequency. The isolated lungs showed significantly lower *W* when compared with supine and prone positions, for both juveniles and adults (Fig. 6).

**Fig. 7.**
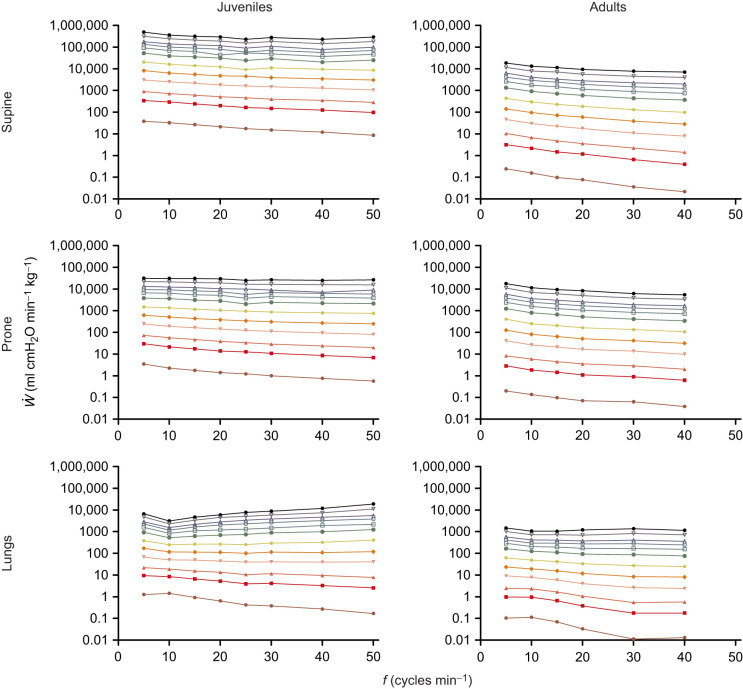
**Relationship between work of breathing (**
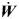
**) and pump frequency at various levels of ventilation in juvenile (left) and adult (right) *C. carbonarius* in supine (top) and prone position (middle) and in isolated lungs (bottom).** Within each graph, the traces from bottom to top represent a ventilation rate of 1, 5, 10, 25, 50, 100, 200, 300, 400, 500, 750 and 1000 ml min^−1^.

We then analyzed minute work of breathing (*Ẇ*). In Fig. 7, we show that, in the intact system and for any given level of ventilation, an increase in frequency led to a decrease in *Ẇ*, while in the isolated lungs, *Ẇ* was more variable, especially in juveniles, decreasing with frequency at lower pump ventilation, but increasing with frequency at greater pump ventilations. *Ẇ* was lowest for the isolated lungs in both juveniles and adults, and in juveniles *Ẇ* was lower in the prone position when compared with the supine one. Position had no effect on *Ẇ* in adults.

## DISCUSSION

While a chelonian in a supine position might not be a common sight, animals occasionally will end up in an inverted position and will need to self-right, which comes at an increased metabolic cost (Ewart et al., 2022; Golubović et al., 2015). Also, chelonians are frequently positioned in a supine position during veterinary practice, fundamentally altering breathing mechanics and consequently demanding adjustments in artificial ventilation to maintain an animal's acid–base and blood gas status (Williams et al., 2021). Overall, we observed that our tested positions (supine and prone) significantly influenced lung mechanics (i.e. *V*_Lr_, *V*_Lm_, *C*_stat_, *C*_dyn_) in both adults and juveniles of *C. carbonarius*. Consistently, lung mechanics in both the supine and prone position significantly differed from that in isolated lungs. As expected, isolated lungs displayed increased *V*_Lr_, *V*_Lm_, *C*_stat_ and *C*_dyn_. Such results indicate an expressive change in respiratory mechanics caused by the limitation of the lungs within body cavity, similar to the observation by Jackson (1971) in the semi-aquatic turtle *T. scripta*.

Regarding *V*_Lr_, our experiment showed that the prone position presents a significant increase in *V*_Lr_ when compared with the supine position. In *T. scripta*, Williams et al. (2021) found a tendency for increased *V*_Lr_ in the prone position with the respiratory system open to the atmosphere, but with no significant difference in the supine position. In *C. carbonarius*, these differences can be explained by the dorsally located lungs in Testudines being impacted by the mass of the viscera when in the supine position. Such an increased load affects breathing mechanics, but the effect may be reduced by the presence of a relatively complete PPS (Perry, 1978; Lambertz et al., 2010), mitigating lung compression (Souza and Klein, 2021; in *T. scripta*). In the prone position, in contrast, gravity will pull the visceral organs away from the lungs, and as the viscera are connected to the PPS (Broman, 1904, 1937), the PPS will be pulled downwards, expanding the space available for the lungs within the carapace, increasing *V*_Lr_. However, a significant difference in *V*_Lm_ might not be expected, as the pressures applied during static lung inflation are great, displacing the viscera in similar manner in the two positions, as has also been shown by Williams et al. (2021) for *T. scripta.* Interestingly, *V*_Lr_ values reported by Williams et al. (2021) are about 3 times greater and the *V*_Lm_ values are 2.5 times greater in *T. scripta* than in *C. carbonarius*, suggesting a significant variation in lung volume among testudine species.

From a functional point of view, the experimentally obtained values for *V*_Lr_ in the supine position might not reflect the *V*_Lr_ in a living terrestrial testudine, as gravity would pull the viscera downwards, increasing *V*_Lr_ as described above. Data on mechanical properties of the respiratory system in terrestrial Testudines might, therefore, be better obtained with animals in the prone position to better reflect the normal expansion of the lungs within the carapace. When it comes to aquatic or semi-aquatic species, however, hydrostatic pressure will act upon the flexible parts of the body wall, pushing the viscera into the body cavity, thereby reducing lung volume (Gaunt and Gans, 1969) and consequently *V*_Lr_, as we observed before (Trevizan-Baú, 2016). Additionally, buoyancy control can significantly alter the volume of the lungs in submerged turtles, as lung volume and bladder volume are intrinsically linked when turtles need to regulate their specific density to maintain a given position within the water column (Jacobs, 1939; 1941; Jackson, 1969, 1971; Peterson and Gomez, 2008). For species breathing while submerged, prone and supine positions might be analyzed to fully understand the mechanical properties of the respiratory system, or ideally might be measured while the animal is submerged (Trevizan-Baú, 2016; Souza and Klein, 2021).

Moreover, differences between juveniles and adults, especially in *V*_Lr_/*V*_Lm_, indicate that a relative difference in visceral mass in adults could exert more pressure on the lungs in a supine position and pull the lungs down more when in a prone position. If this is the case, visceral pressure would be at least partially responsible for the differences observed between juveniles and adults in the intact system. However, when the viscera were removed, juveniles exhibited greater lung compliance compared with adults. This suggests a potentially more distensible lung in the juvenile life stage. Consequently, other hypotheses could be related to these factors: (i) because of the lesser ossification of the carapace and by standardizing data by *M*_B_, juvenile compliance values were consistently greater when compared with those of adults; and (ii) ontogenetic differences in the lungs and/or PPS between juveniles and adults could also influence compliance. Either way, a more detailed investigation of the function of the PPS and/or changes during ontogenetic development in the present and in other testudine species seems warranted.

Following Pages et al. (1990), we also normalized our data by pulmonary volume (i.e. *V*_Lr_ and *V*_Lm_), in addition to *M*_B_. These normalizations could avoid the effect of physiological or nutritional factors that may occur when normalizing by *M*_B_ and furthermore explain whether differences in breathing mechanics might be related to differences in respiratory system volume or mechanical properties of the respiratory system (York et al., 2018). Our comparisons between *C*_stat_ standardized by pulmonary volume indicate fewer changes between juveniles and adults when compared with *C*_stat_ standardized by *M*_B_. In addition, a decreased slope in regressions when *C*_stat_ was standardized by pulmonary volume may indicate an influence of *M*_B_ standardization on the results, possibly as a result of differences in shell ossification (Oliveira et al., 2023).

Differences in relative lung volume (*V*_Lr_/*V*_Lm_), with juveniles presenting a relatively larger lung volume than supine adults, could indicate a possible ontogenetic aspect that explains differences in lung static compliance. Similar to our observation, Reichert et al. (2019) found a higher *C*_stat_ in juveniles compared with adult *C. yacare*. Presumably, the stiffness of the body wall in adult caimans leads to a less compliant system. In *C. carbonarius*, juveniles possess a less ossified carapace compared with adults (Oliveira et al., 2023). Thus, this could influence the greater total compliance found in juveniles. However, our *C*_dyn_ results indicate a similarity in *C*_L_ between the groups. We only conducted a one-volume comparison between juveniles and adults and only from 5 to 20 cycles min^−1^, so more *C*_dyn_ comparisons between proportional lung volumes must be done to fully understand a possible ontogenetic factor that could influence lung compliance.

The combinations of frequencies and volumes used by the animals can be a strategy they use to minimize the cost of breathing. In our experimental animals, we observed that volume influences both *C*_dyn_ and *W* more than frequency does. As depicted in Fig. 5, frequency increase exhibited greater stability in *C*_T_ compared with a pronounced decrease in *C*_L_ and with a moderated decrease in *C*_B_. For *T. scripta*, Vitalis and Milsom (1986b) found that, as frequency increases, the work required to inflate the lungs contributes more to the total work of the system. The authors indicated that both static and dynamic mechanics, especially at lower frequencies, are influenced more by the body wall, rather than the lungs, and the increase in lung work with increasing frequency results from an escalation in non-elastic forces required to overcome ventilation (Vitalis and Milsom, 1986b).

As reported in previous studies, the combination of frequency and volume plays a crucial role in meeting respiratory demands while incurring minimal breathing costs. Oliveira et al. (2023) observed, under normoxic conditions, an elevation in instantaneous breathing frequency (*f*′, breaths min^−1^) despite a constant tidal volume (*V*_T_, ml), when both juveniles and adults were exposed to an increase in temperature (15–35°C). Additionally, Trevizan-Baú et al. (2018) found that adults exhibited heightened ventilation characterized by increased volume and frequency when exposed to hypoxic and hypercarbic environments.

Work of breathing (*W*) seems to be greater in juveniles when compared with adults, but this is due to the greatly different *M*_B_ of the two groups and the different pump volumes used. To better compare the applied pump volumes, we divided (as we did for *C*_dyn_; Fig. 5) pump volume by juvenile and adult *M*_B_. According to this calculation, the 0.6 ml pump volume of juveniles is proportional to the 25 ml pump volume of adults, as these volumes represent *M*_B_-corrected pump volumes of ∼7.6 ml kg^−1^. Comparing these two pump volumes reveals that *W* in supine adults is almost double (43.0–50.9 ml cmH_2_O min^−1^ kg^−1^) that in juveniles in the supine position (26.8–33.5 ml cmH_2_O min^−1^ kg^−1^). This difference remains when animals are in the prone position (juveniles: 26.1–33.9 ml cmH_2_O min^−1^ kg^−1^; adults 48.6–56.0 ml cmH_2_O min^−1^ kg^−1^), but not in the isolated lungs (juveniles: 7.6–10.9 ml cmH_2_O min^−1^ kg^−1^; adults 8.0–13.7 ml cmH_2_O min^−1^ kg^−1^) (Fig. 6). This greater *W* at proportional pump volumes can be attributed to the greater *C*_dyn_ seen in supine and prone juveniles when compared with adults (Fig. 4). Surprisingly, the greater *C*_dyn_ of isolated juvenile lungs did not translate into lower *W* when compared with adult isolated lungs.

Juvenile *C. carbonarius* individuals show a minute ventilation of about 6 ml min^−1^ kg^−1^ at 25°C and about 10 ml min^−1^ kg^−1^ at 35°C, while adults display greater levels of ventilation at both temperatures (∼20 ml min^−1^ kg^−1^ at 25°C and ∼60 ml min^−1^ kg^−1^ at 35°C, respectively) (Oliveira et al., 2023). Exposing adult *C. carbonarius* to hypoxia or hypercarbia, Trevizan-Baú et al. (2018) found that ventilation increased up to 150 ml min^−1^ kg^−1^ at 25°C. Only the lowest level of ventilation (1 ml min^−1^; Fig. 7) chosen in the present study reflects minute ventilation seen in resting juvenile *C. carbonarius* as it represents a *M*_B_-corrected ventilation of 12.5 ml min^−1^ kg^−1^. Regarding adults, the ventilation volumes of 10, 25, 50, 100, 200, 300, 400 and 500 ml min^−1^ represent *M*_B_-corrected ventilation volumes of 3.1, 7.7, 15.4, 30.7, 61.5, 92.3, 123.1 and 153.8 ml min^−1^ kg^−1^, respectively, values that cover the physiological range of minute ventilation seen in adult animals well. While 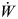 was lowest for the isolated lungs in both groups, position showed a more pronounced effect in juveniles when compared with adults. Juveniles showed a greater 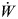 in the supine position, which could be expected from the viscera resting on the lungs while being in an inverted position. Once this impact was removed in the prone position, 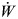 decreased for all levels of ventilation and frequency. In adults, however, 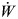 was very similar in the two body positions, which seems surprising given that the absolute visceral mass resting on the adult lungs while in a supine position should be significant. An explanation for the similar 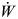 could be the presence of a well-developed PPS in combination with a well-ossified shell. In adults, a firm PPS separating the lungs from the remaining viscera while being attached to an immobile carapace might avoid a significant impact of the viscera while in an inverted position. In juveniles, in contrast, the carapace is less ossified and still flexible (Oliveira et al., 2023) and the PPS might be less developed, thereby increasing the effect of the visceral mass on the lungs when in a supine position. A detailed examination of the development of the PPS in this chelonian combined with its effect on breathing mechanics seems necessary to better understand the differences observed by us between juvenile and adult *C. carbonarius*.

### Conclusion

*Chelonoidis carbonarius* shows some differences in mechanical properties, when comparing prone with supine positions. These differences can be explained by a certain influence of the viscera on the lungs. However, the complete post-pulmonary septum present in these animals could reduce the pressure of the viscera when animals are in the supine position. The isolated lungs, as expected, showed a large compliance and, consequently, a decreased work of breathing when compared with the intact system. Combinations of volume and frequency indicate that mechanics are more volume dependent, corroborating data published on the lung ventilation of this species. Unlike semi-aquatic species, such as *T. scripta*, which are more frequency dependent, this observation may be associated with the terrestrial habitat of the living animals. The differences found between juveniles and adults may be affected by the normalization of the lung volume (e.g. *M*_B_ versus *V*_Lr_), especially given the less ossified carapace in juveniles. Nevertheless, juveniles showed a larger relative lung volume than adults, indicating a possible large lung volume related to size in the early stages of life in *C. carbonarius*.
